# Threshold-dependent iodine imaging and spectral separation in a whole-body photon-counting CT system

**DOI:** 10.1007/s00330-021-07786-0

**Published:** 2021-03-13

**Authors:** S. Sawall, L. Klein, E. Wehrse, L. T. Rotkopf, C. Amato, J. Maier, H.-P. Schlemmer, C. H. Ziener, S. Heinze, M. Kachelrieß

**Affiliations:** 1 Division of X-Ray Imaging and CT, German Cancer Research Center (DKFZ), Im Neuenheimer Feld 280, 69120 Heidelberg, Germany; 2 Medical Faculty, Ruprecht-Karls-University Heidelberg, Im Neuenheimer Feld 672, 69120 Heidelberg, Germany; 3 Department of Physics and Astronomy, Ruprecht-Karls-University Heidelberg, Im Neuenheimer Feld 226, 69120 Heidelberg, Germany; 4 Division of Radiology, German Cancer Research Center (DKFZ), Im Neuenheimer Feld 280, 69120 Heidelberg, Germany; 5 Institute of Forensic and Traffic Medicine, University Hospital Heidelberg, Voßstraße 2, 69115 Heidelberg, Germany

**Keywords:** Tomography, X-ray computed, Contrast media, Angiography, Iodine, Phantoms, imaging

## Abstract

**Objective:**

To evaluate the dual-energy (DE) performance and spectral separation with respect to iodine imaging in a photon-counting CT (PCCT) and compare it to dual-source CT (DSCT) DE imaging.

**Methods:**

A semi-anthropomorphic phantom extendable with fat rings equipped with iodine vials is measured in an experimental PCCT. The system comprises a PC detector with two energy bins (20 keV, *T*) and (*T*, e*U*) with threshold *T* and tube voltage *U*. Measurements using the PCCT are performed at all available tube voltages (80 to 140 kV) and threshold settings (50–90 keV). Further measurements are performed using a conventional energy-integrating DSCT. Spectral separation is quantified as the relative contrast media ratio R between the energy bins and low/high images. Image noise and dose-normalized contrast-to-noise ratio (CNRD) are evaluated in resulting iodine images. All results are validated in a post-mortem angiography study.

**Results:**

R of the PC detector varies between 1.2 and 2.6 and increases with higher thresholds and higher tube voltage. Reference R of the EI DSCT is found as 2.20 on average overall phantoms. Maximum CNRD in iodine images is found for *T* = 60/65/70/70 keV for 80/100/120/140 kV. The highest CNRD of the PCCT is obtained using 140 kV and is decreasing with decreasing tube voltage. All results could be confirmed in the post-mortem angiography study.

**Conclusion:**

Intrinsically acquired DE data are able to provide iodine images similar to conventional DSCT. However, PCCT thresholds should be chosen with respect to tube voltage to maximize image quality in retrospectively derived image sets.

**Key Points:**

*• Photon-counting CT allows for the computation of iodine images with similar quality compared to conventional dual-source dual-energy CT.*

*• Thresholds should be chosen as a function of the tube voltage to maximize iodine contrast-to-noise ratio in derived image sets.*

*• Image quality of retrospectively computed image sets can be maximized using optimized threshold settings.*

## Introduction

Over the last decades, computed tomography (CT) has proven to be an invaluable tool in clinical diagnostics. State-of-the-art systems allow for the acquisition of whole-body patient data in seconds and the resulting reconstructions provide a spatial resolution in the order of less than a millimeter. Besides conventional single energy imaging based on the integrated information from a single x-ray spectrum, dual-energy CT (DECT) techniques have been introduced to clinical practice extending diagnostic capabilities even further. Popular applications include the computation of virtual monochromatic (VMI) and virtual non-contrast (VNC) images or material characterization and decomposition [1–3]. Various techniques have been proposed and are currently used in clinical CT systems to realize DECT. Most commonly, DECT is performed by using two distinct source-detector-pairs, rapid kV-switching, dual-layer sandwich detectors, or split filters [4–7]. Recently, prototypes of novel photon-counting (PC) detectors became available extending the range of dual- and multi-energy acquisition strategies. In the case of energy-integrating (EI) detectors, an incoming x-ray photon is absorbed in the scintillator, typically Gd_2_O_2_S, resulting in the emission of optical photons that are detected in photo diodes eventually forming the desired signal. In the case of photon-counting detectors, the scintillator is replaced by a semiconductor, usually cadmium telluride (CdTe). The absorption of an incoming x-ray photon results in the formation of a charge cloud that is transported to electrodes that are constituting the different detector pixels using a bias voltage in the order of 1 kV [8–10]. The formed signals allow not only for the counting of single photons but also for the quantification of their energy. Typically, x-ray photons are sorted into two to four bins according to their energy, facilitating dual- or multi-energy acquisitions. In the case of two energy bins, a single threshold is used and photons with energy below this threshold are sorted to the lower bin and photons with energy above this threshold are sorted to the upper bin. This threshold can usually be placed almost arbitrarily with respect to the detected x-ray spectrum and influences the image quality of derived image sets, e.g., iodine maps or VMIs. Since the detectors always acquire at least two energy bins, spectral data are available even if no dedicated dual-energy acquisition was performed. A retrospective dual-energy evaluation of the acquired data might be desired, e.g., in the case of incidental findings or to retrospectively improve contrast agent enhancement. Thus, thresholds should always be set to maximize image quality even if a DECT evaluation is not within the initial scope of the examination. Hence, the influence of threshold position on the image quality of derived image sets, e.g., the iodine material maps, is of high interest and should be prospectively considered when setting up scan protocols [11]. This optimization problem shall be evaluated in this work. Hence, our focus is on contrast-enhanced imaging. The optimal threshold positions for other applications, e.g., virtual non-calcium images or imaging of potential other contrast agents [12, 13], might differ given the spectral properties of the materials of interest. In particular, an experimental CT system equipped with a prototype photon-counting detector is used in all studies presented here. This detector has already proven to allow for dual- and multi-energy decomposition of conventional and potential novel contrast agents, to provide iodine quantification with high accuracy, and to allow for the computation of VMIs, among others, and several contrast-enhanced patient studies have been published [14–20]. We here evaluate the quality of derived iodine maps as a function of threshold, patient size, and tube voltage in a phantom study. Since the ionizing nature of x-rays prohibits multiple examinations of patients, the results will be verified in a post-mortem CT angiography study.

## Materials and methods

### CT system

**Fig. 1:**
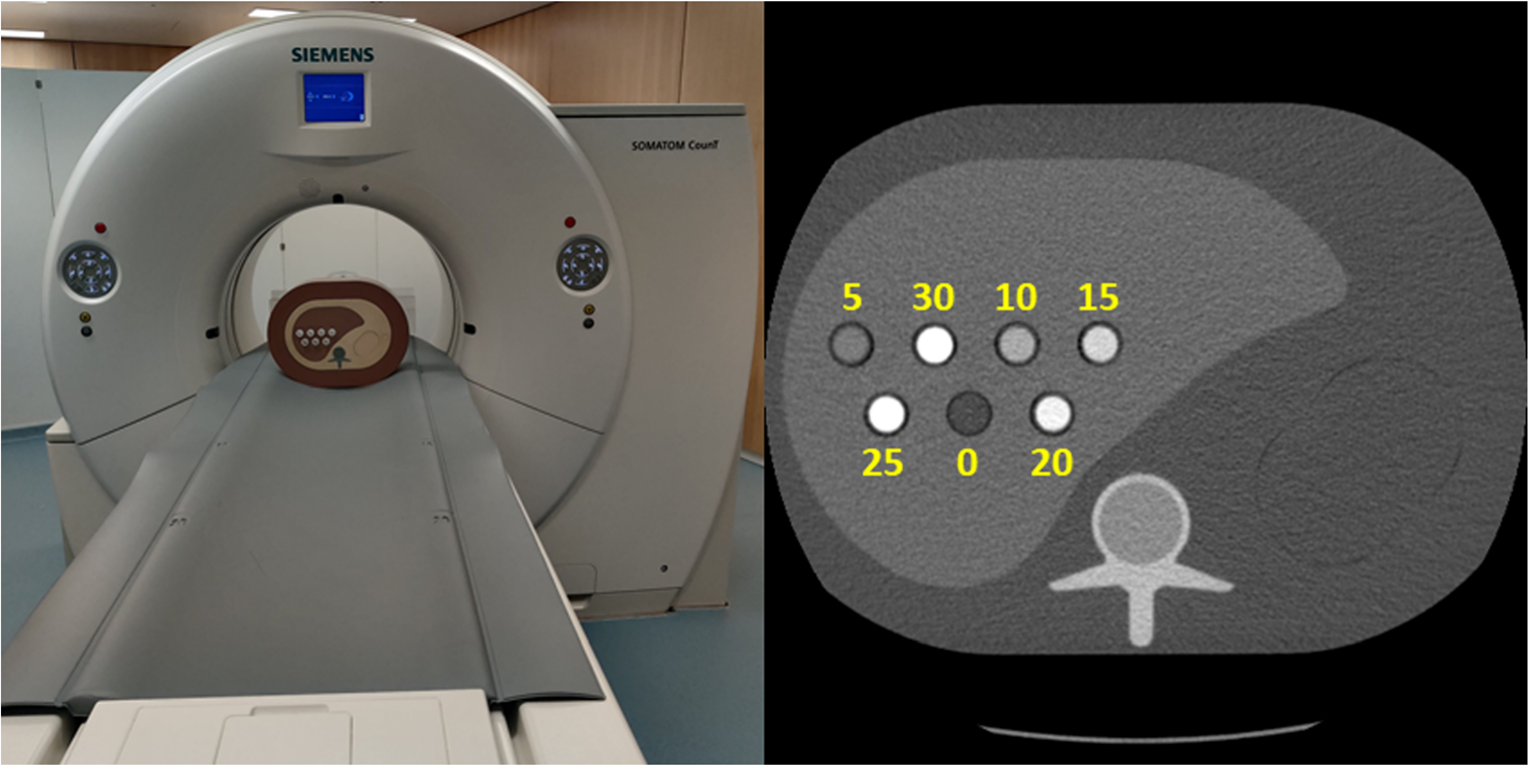
Left: Experimental photon-counting CT and the used semi-anthropomorphic phantom. Right: Semi-anthropomorphic liver phantom used in the experiments. The numbers indicate the iodine concentration in the vials measured in mg/mL. Zero denotes pure water

Our experiments have been performed using the SOMATOM CounT experimental CT system (Siemens Healthineers, Fig. 1 left). This system is based on the SOMATOM Definition Flash dual-source CT whereas one of the conventional EI detectors was replaced by a PC detector allowing for a comparison of both technologies in a single system. The PC detector provides a native pixel size of 225 μm but is operated in 4 × 4 binning mode referred to as “macro.” This results in a pixel size of 0.50 mm in the iso-center which is close to the size of the energy-integrating detector of 0.6 mm. Hence, the macro mode is the standard mode of operation provided by the experimental system. Measurements using the PC detector provide a field of measurement (FOM) of 275 mm while the EI detector in this system allows for acquisitions with a FOM of 500 mm. Hence, patients and phantoms might exceed the FOM of the PC detector and a data completion scan using the EI detector is required to prevent degradation of image quality by truncation artifacts. This data completion scan would contribute to the overall dose. However, recent experiments illustrated that the required dose is similar to a single chest x-ray [21]. Furthermore, the clinical version of a photon-counting CT might provide a sufficiently sized detector array and hence the data completion scan will be disregarded in the following. Unlike the EI detector, an operation of the PC detector in macro mode results in the acquisition of multi-energy data. In particular, the detector simultaneously acquires two energy bins with the lower bin covering an energy range of (*T*_1_, *T*_2_) and the higher bin covering (*T*_2_, *eU*), with *U* being the tube voltage and *e* being the elementary charge, while also providing images covering the whole spectrum, i.e., (*T*_1_, *eU*). Reconstructed images of the energy bins will be referred to as *f*_Low_ for the lower bin and *f*_High_ for the upper bin, whereas *f* denotes the image reconstructed using the entire spectrum. The thresholds *T*_1/2_ can be chosen almost arbitrarily between 20 and 50 keV for *T*_1_, and 50 and 90 keV for *T*_2_. The threshold *T*_1_ will be fixed at 20 keV, and hence, *T*_2_ will only be referred to as *T* in the following.

### Material decomposition

Let us consider CT images normalized to air at 0 and water at 1 [22]. Given the reconstructed image of the lower energy bin *f*_Low_ and the image of the high energy bin *f*_High_, one might compute a water image *f*_W_, i.e., a VNC image, as

1 $$ {f}_W=\left(1-\beta \right){f}_{\mathrm{Low}}+\beta {f}_{\mathrm{High}} $$

Here, β = *R*/(*R*-1) is a weighting coefficient wherein *β* = *β*(*U*, *T*) is a function of the tube voltage *U* and threshold *T*. The scalar *R* is the relative contrast media ratio (RelCM) and a measure for the spectral separation between the images *f*_Low_ and *f*_High_ [23]_._ It is sometimes also referred to as CT number ratio or dual-energy ratio [24]. I.e., given two water-iodine mixtures of unknown mixing ratio, one can show that *R* is independent of the mixing ratio and is only a function of the CT values of iodine in these images as [22]

2 $$ R={CT}_{\mathrm{Low}}/{CT}_{\mathrm{High}} $$

In practice, *CT*_Low_ and *CT*_High_ are the mean CT values measured in regions of interest containing diluted contrast media, e.g., the aorta or portal vein, and can be used to calibrate DECT. The variance of *f*_W_ is

3 $$ \mathrm{Var}\ {f}_W={\left(1-\beta \right)}^2\mathrm{Var}\ {f}_{\mathrm{Low}}+{\beta}^2\mathrm{Var}\ {f}_{\mathrm{High}}. $$

The noise in the VNC image is thus ${\sigma}_{f_W}=\sqrt{\mathrm{Var}{f}_W\ }$ which is a function of the variance and thus of the noise in *f*_Low_ and *f*_High_. Similar to a VNC image, an iodine image *f*_I_ can be computed as

4 $$ {f}_I=\gamma \left({f}_{\mathrm{Low}}-{f}_{\mathrm{High}}\right). $$

The variance of this iodine map is

5 $$ Var\ {f}_I={\gamma}^2\mathrm{Var}\ {f}_{\mathrm{Low}}+{\gamma}^2\mathrm{Var}\ {f}_{\mathrm{High}}. $$

The scalar *γ* is chosen such that *f*_I_+*f*_W_=f_α_ with *f*_α_ being a mixed image with minimum noise [25], i.e.,

6 $$ {f}_{\alpha }=\left(1-\alpha \right){f}_{\mathrm{Low}}+\alpha {f}_{\mathrm{High}}. $$

The scalar *α* = Var *f*_Low_/(Var *f*_Low_ + Var *f*_High_) is chosen according to an inverse variance weighting that minimizes the noise in the resulting image *f*_α_. Given its dependence on *f*_α_ and *f*_W_, the iodine image is also a function of tube voltage, threshold, and noise in *f*_Low_ and *f*_High_. In the case of conventional dual-source dual-energy imaging, the noise in *f*_Low_ and *f*_High_ can be adapted separately by adjusting the tube current for the two different x-ray sources. *R* remains unaffected as long as the tube voltages are unaltered. Hence, the optimal image in terms of noise and CNRD is found for balanced noise levels between both tubes. In the case of photon-counting CT with a single detector, noise in *f*_Low_ and *f*_High_ is a function of the threshold and cannot be adjusted separately. Furthermore, changing the threshold changes not only the image noise but also the detected spectrum in each bin and hence *R*. Thus, the lowest noise in the iodine image might not be found for balanced noise levels between both bins.

Note that in practice, *f*_W_ and *f*_I_ usually undergo a variety of vendor-specific post-processing steps to compensate for the increase in noise due to the material decomposition. However, only the raw material maps prior to any post-processing shall be evaluated and presented in the following to exclude any potential benefits or drawbacks of the used de-noising methods.

### Phantom experiments

All phantom experiments presented in the following have been performed using a semi-anthropomorphic liver phantom (QRM) that can be extended with fat rings to mimic different patient sizes. In particular, a small-sized (S, 20 cm × 30 cm), a medium-sized (M, 25 cm × 35 cm), and a large-sized patient (L, 30 cm × 40 cm) were modelled. All phantoms were equipped with vials containing iodine solutions with concentrations between 5 and 30 mg/mL (see Fig. 1). Image acquisition was performed using all available tube voltages, namely 80 kV, 100 kV, 120 kV, and 140 kV with a constant dose level *D* of 11 mGy (CTDI_32cm_) allowing for a comparison of results at unit dose. The detector’s energy threshold was varied in steps of 5 keV between 50 keV and the maximum available threshold for a respective tube voltage. In particular, the maximum available thresholds were 70 keV for a tube voltage of 80 kV, 80 keV for a tube voltage of 100 kV, and 90 keV for tube voltages of 120 kV and 140 kV, respectively. Image reconstruction was performed using weighted filtered backprojection covering the entire FOV of 275 mm using a D40f-kernel onto a matrix of 512 × 512 pixels resulting in a pixel size of 0.54 mm and with a slice thickness of 1.50 mm and a slice increment of 0.75 mm. *R* was evaluated using ROIs placed in the iodine insert containing 15 mg/mL. Iodine images were computed according to Eq. (4) and the contrast-to-noise ratio at unit dose (CNRD) was measured as

7 $$ \mathrm{CNRD}=\frac{\left|{c}_1-{c}_2\right|}{\sqrt{\sigma_1^2+{\sigma}_2^2}}\bullet \frac{1}{\sqrt{D}}. $$

Therein, *c*_i_ are iodine concentrations measured in the 15 mg/mL iodine insert and the background and *σ*_*i*_ are the corresponding standard deviations measured in these ROIs. Since all acquisitions have been performed at the same dose level, a normalization of the CNR with respect to dose is not required. However, we prefer to report CNRD to illustrate that it is a function of dose in general. To investigate the influence of image noise in general and the balancing of noise between individual bins, we define the noise quotient (*RelNoise*) as follows:

8 $$ \mathrm{RelNoise}=\frac{\sigma_{\mathrm{Low}}}{\sigma_{\mathrm{High}}}. $$

Reference measurements were conducted using an energy-integrating dual-source dual-energy CT system (Definition Flash) using tube voltage pairs of 80 kV/Sn140 kV and 100 kV/Sn140 kV with Sn denoting an additional 0.4-mm tin prefilter to improve the spectral separation. The tube currents were adjusted to match the radiation dose level used at the photon-counting CT. The FOM of the DSCT is 500 mm and 330 mm for the first and second imaging chains, respectively. To match the pixel size of reconstructions obtained using the PC detector, reconstructions of the DSCT cover a FOV of 275 mm. A summary of all relevant acquisition and reconstruction parameters can be found in Table 1.

**Table 1:** Acquisition and reconstruction parameters for all experiments conducted with phantoms and the post-mortem angiography study (*FOM*, field of measurement; *FOV*, field of view; *AEC*, automatic exposure control)

	DSCT	PCCT
Tube voltage_phantoms_	80 kV/140 kV + Sn, 100 kV/140 kV + Sn	80 kV, 100 kV, 120 kV, 140 kV
Tube voltage_post-mortem_	-	140 kV
CTDI_32 cm_	11 mGy	11 mGy
AEC	off	off
FOM	500 mm/330 mm	275 mm
FOV	275 mm	275 mm
Image matrix	512 × 512 px	512 × 512 px
Pixel size	0.54 mm	0.54 mm
Slice thickness	1.50 mm	1.50 mm
Slice increment	0.75 mm	0.75 mm
Kernel	D40f	D40f

### Post-mortem CT angiography study

Given the ionizing radiation used in CT, experiments similar to the phantom measurements cannot be performed in patients since multiple acquisitions would be required. To overcome this drawback and to illustrate that the results obtained using phantoms can be translated to clinical practice, a post-mortem CT angiography study was performed after approval by the local ethics committee (S-021/2020). This study follows published and accepted protocols [26, 27]. In brief, a cadaver was placed in supine position on the patient table and the right femoral vessels were cannulated. About 3.5 L of an iodine-based contrast medium was injected with a flow rate of about 800 mL/min. The oily nature of the contrast agent (Angiofil-Macro, Fumedica AG and paraffin oil) and its optimized viscosity prevent a high loss of perfusate into the surrounding tissue and are reported to prevent rapid tissue edematization [28]. Images were acquired using a tube voltage of 140 kV and a dose of 11 mGy using the PC detector. The threshold was varied between 50 and 90 keV in steps of 5 keV. Iodine images were computed according to Eq. 4. Comparative acquisitions at the DSCT similar to the phantom experiments could not be conducted in the presented post-mortem study due to the contrast agent kinetics. I.e., a transport of the cadaver to the DSCT after PC acquisitions, increasing extravasation of contrast media over time, and an accumulation of contrast media at the bottom of vessels due to the lack of circulation alter the distribution of contrast media significantly and do not allow for a fair side-by-side comparison of both technologies as shown in the phantom experiments.

## Results

### Spectral separation

**Fig. 2:**
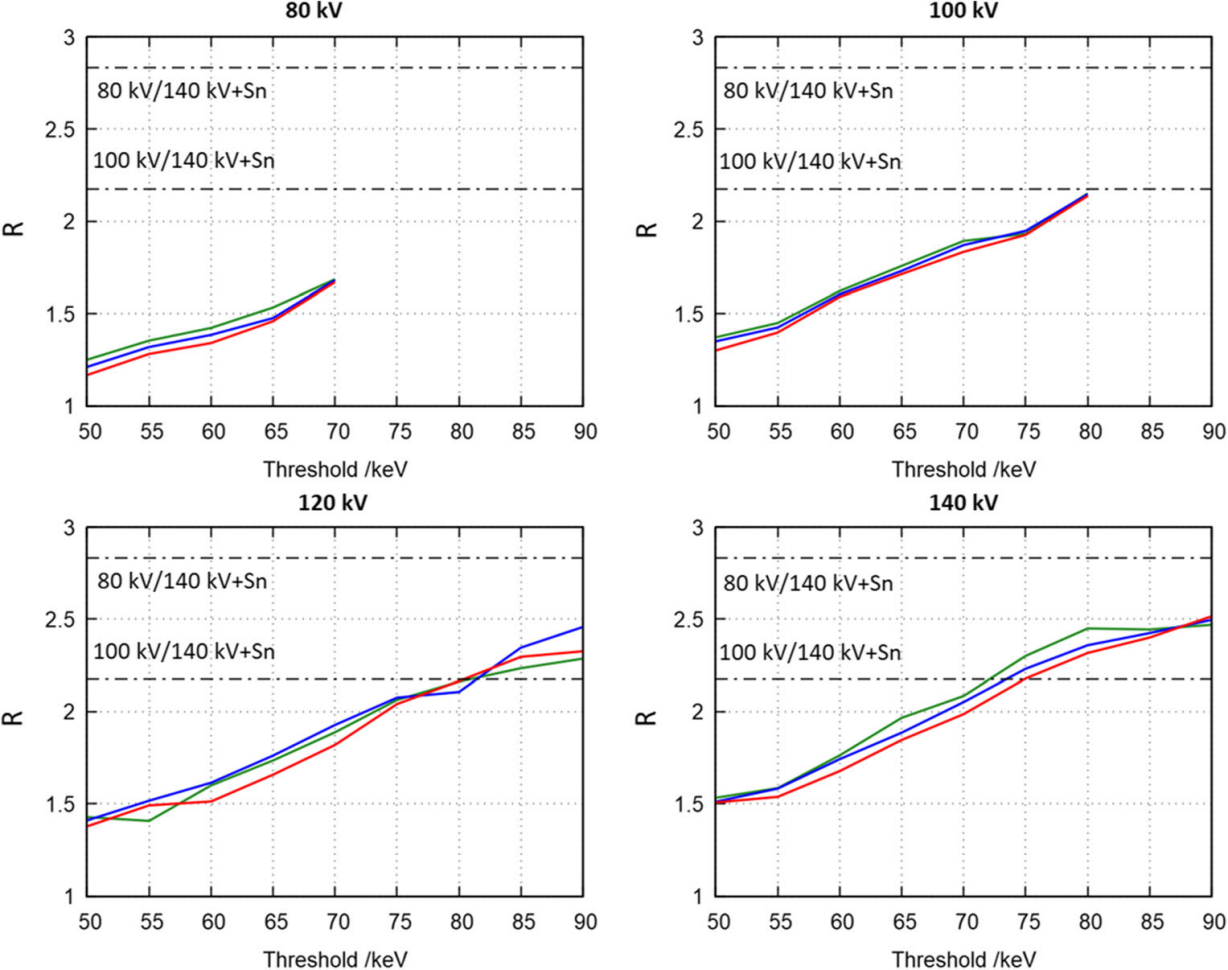
*R* as function of threshold for all available tube voltages. Colors encode phantom sizes, i.e., the S-phantom (green), the M-phantom (blue), and the L-phantom (red). Black dashed-dotted lines show *R* measured in a reference dual-source dual-energy CT system and represent the average overall phantom sizes

Figure 2 shows *R* for all available tube voltages obtained in the insert containing 15 mg I/mL. Colored, solid lines in this figure represent different phantom sizes, i.e., the S-phantom (green), the M-phantom (blue), and the L-phantom (red). For each tube voltage, the threshold was varied in steps of 5 keV between 50 keV and the maximum possible value for the respective tube voltage. Black dashed-dotted lines show *R* measured in an energy-integrating, dual-energy CT and represent the average overall phantom sizes. In the case of the photon-counting detector, *R* steadily increases with an increasing threshold. E.g., in the case of 140 kV, *R* is about 1.5 for a threshold of 50 keV and rises to about 2.5 at a threshold of 90 keV. This holds for all investigated tube voltages. At any given threshold, *R* increases with increasing tube voltage. E.g., for a threshold of 60 keV, a tube voltage of 80 kV results in a *R* of about 1.4, and in 1.6 using 100 kV, 1.65 for 120 kV and finally in 1.76 using 140 kV. The reference DECT system achieves relative contrast media ratios of about 2.8 for 80 kV/Sn140 kV and about 2.2 for 100 kV/Sn140 kV which is consistent with values already published in the literature [23]. Hence, the reference system generally shows a higher relative contrast media ratio compared to the investigated photon-counting detector. Only higher thresholds and tube voltages of 120 kV and 140 kV, respectively, exhibit similar values to the 100 kV/Sn140 kV reference protocol.

### Material decomposition

**Fig. 3:**
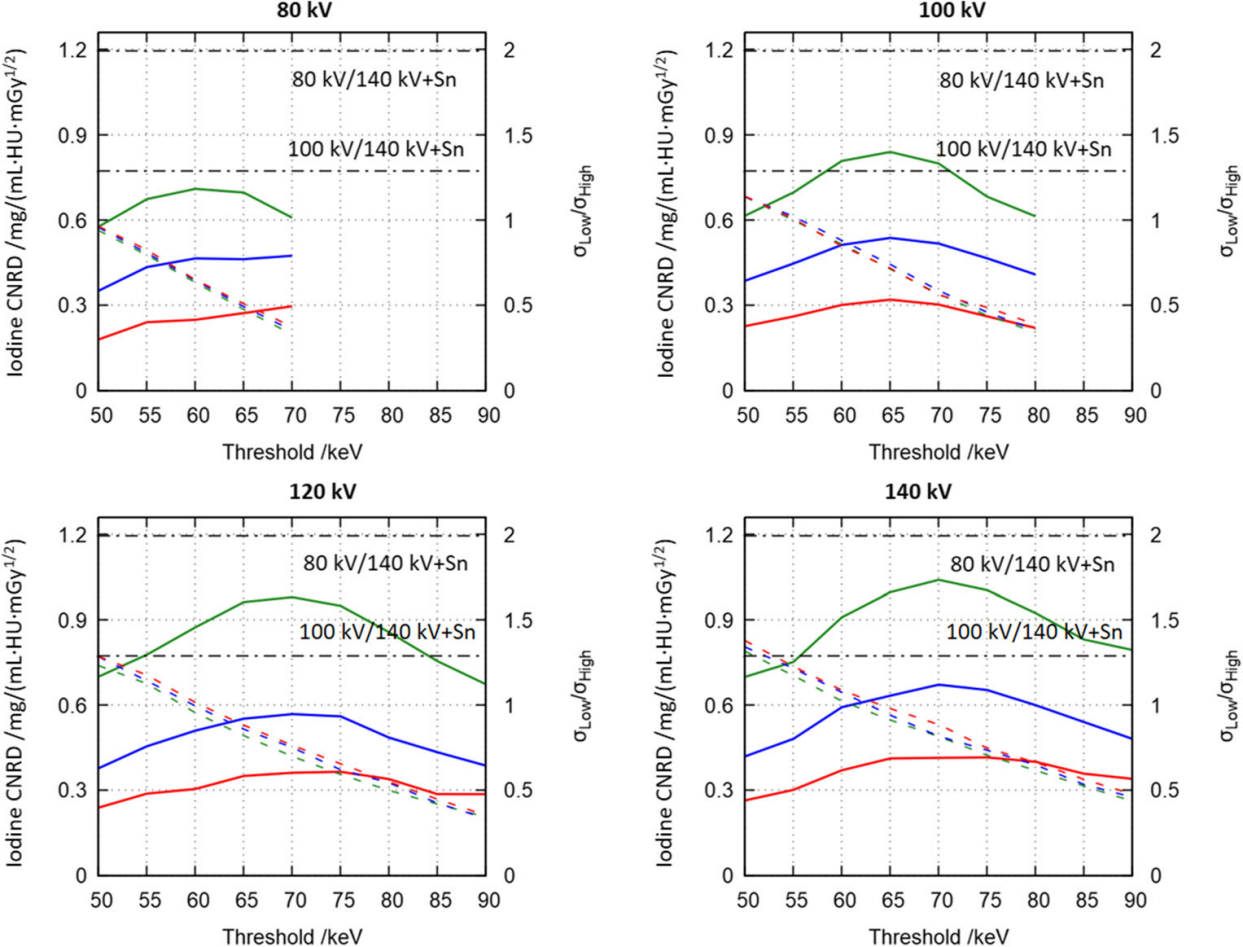
Dose-normalized contrast-to-noise ratio (CNRD) of iodine maps (colored solid lines) and noise quotient (*RelNoise* = σ_Low_/σ_High_, dashed lines) for all phantoms sizes, all tube voltages, and all thresholds. Colors encode phantom sizes, i.e., the S-phantom (green), the M-phantom (blue), and the L-phantom (red). Black dashed-dotted lines were acquired using the M-phantom in a reference dual-source dual-energy CT system

Figure 3 shows the CNRD (see Eq. 7, colored solid lines) for all available tube voltages using thresholds varied in 5-keV steps similar to Fig. 2. The line colors encode the phantom sizes. Black solid lines show the CNRD obtained using the reference DECT system using the S-phantom. Furthermore, the figure shows *RelNoise* (see Eq. 8, dashed lines). In general, CNRD decreases with increasing phantom size and increasing intersection lengths, due to the increase in image noise. All CNRD plots obtained using the investigated photon-counting detector show distinct maxima. In the case of the S-phantom, the optimal CNRD using a tube voltage of 80 kV is found as 0.71 mGy^-1/2^ at a threshold of 60 keV. For 100 kV, the optimal CNRD of 0.84 mGy^-1/2^ is achieved at a threshold of 65 keV and tube voltages of 120 kV and 140 kV show maxima at 70 keV with CNRDs of 0.98 mGy^-1/2^ and 1.04 mGy^-1/2^, respectively. While CNRD decreases with increasing phantom size, the position of the optimal threshold remains unchanged. This holds for all investigated tube voltages and all phantom sizes. The noise quotient *RelNoise* illustrates that unlike conventional DECT, maximum CNRD is not found for balanced background noise levels between bins. In particular, maximum CNRD is found for a noise quotient of 0.63 in the case of 80 kV, 0.72 for 100 kV, 0.73 for 120 kV, and 0.74 for 140 kV, respectively. The noise quotient does not show a dependence on phantom size. As indicated in Fig. 2, the used reference system achieves higher CNRDs of 1.29 mGy^-1/2^ and 1.99 mGy^-1/2^ for the protocols 80 kV/Sn140 kV and 100 kV/Sn140 kV. Previous studies illustrated that the root-mean-square errors between ground truth iodine concentrations and the ones measured in material maps are similar between the reference DECT system and the experimental system equipped with a photon-counting detector [11].

**Fig. 4:**
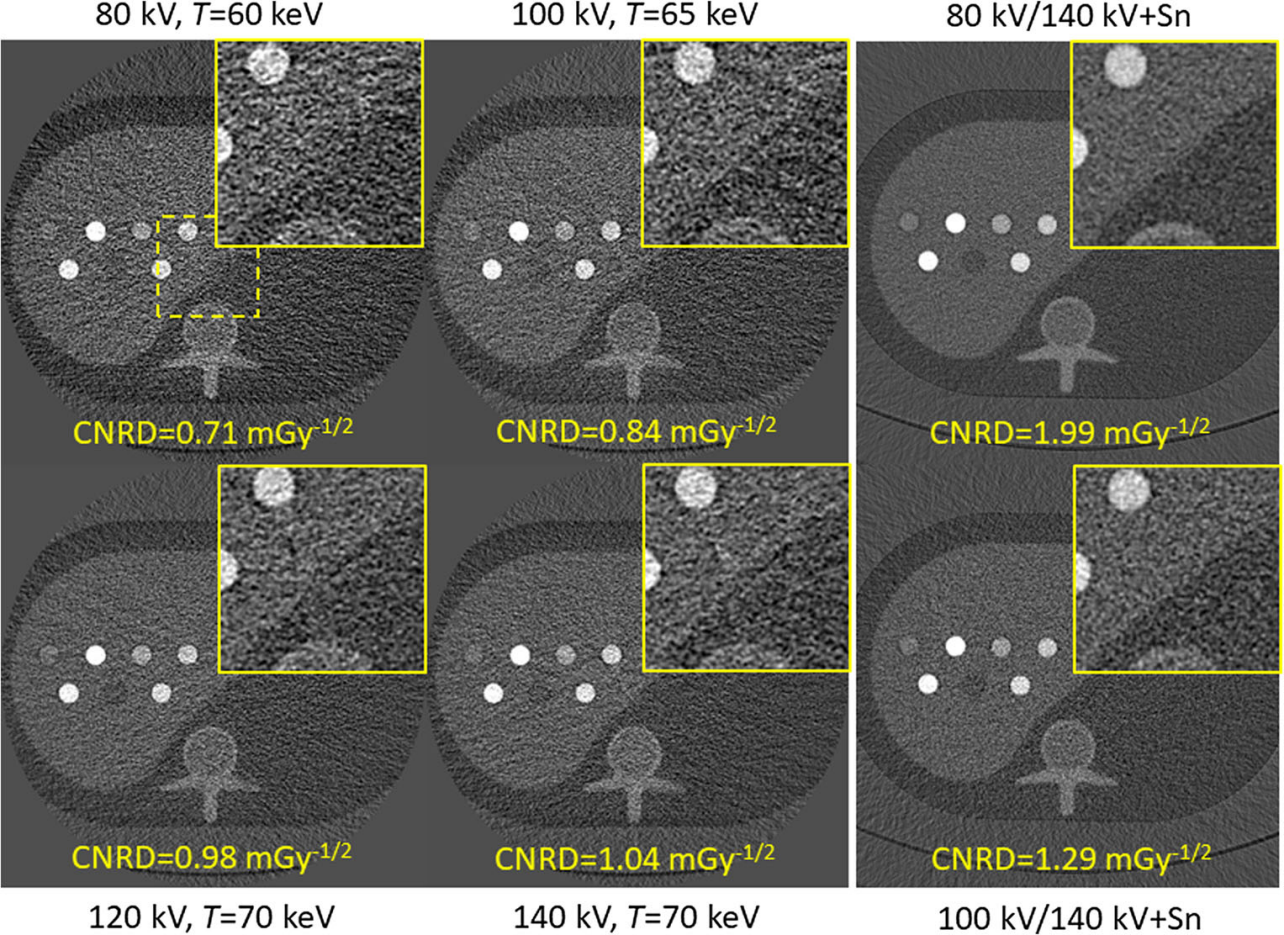
Iodine maps for all available tube voltages of the S-phantom acquired at the same dose. Images were acquired at the optimal threshold according to Fig. 3. Magnifications of the yellow dashed region of interest are shown in the top right of each figure (*C* = 7 mg/mL, *W* = 30 mg/mL)

The results presented in Fig. 3 are further visualized in Fig. 4. The figure shows iodine material maps obtained of the S-phantom for all available tube voltages acquired at the optimal threshold and acquired at the same dose level. It is evident that acquisitions and material decomposition with 80 kV result in the highest noise among all acquisitions. The lowest noise and highest CNRD for the photon-counting detector are found at 140 kV and using a threshold of 70 keV.

### Post-mortem CT angiography study

**Fig. 5:**
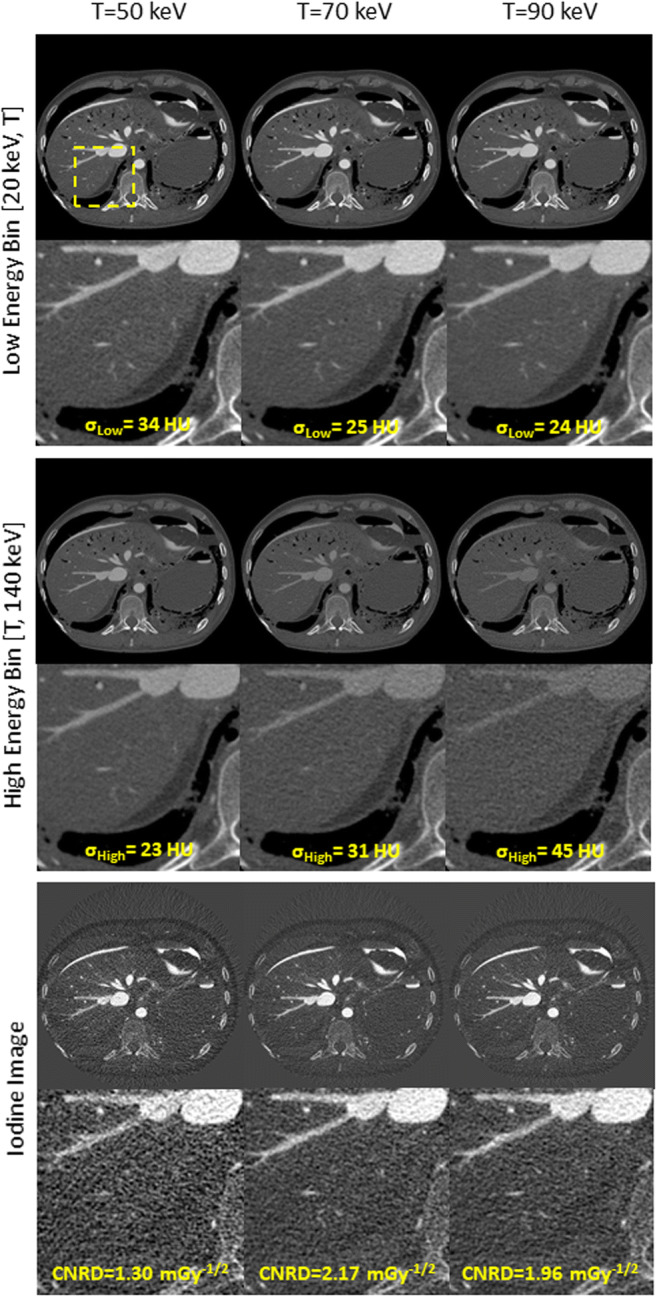
Post-mortem CT angiography using a tube voltage of 140 kV for thresholds of 50 keV (left column), 70 keV (middle column), and 90 keV (right column). Low energy bins (top row), high energy bins (middle row), and iodine maps (bottom row) (bin images *C* = 300 HU, *W* = 1400 HU; material maps *C* = 15 mg/mL, *W* = 30 mg/mL)

To illustrate that the phantom results can be translated to clinical practice, Fig. 5 shows reconstructions obtained in a post-mortem CT angiography study. The figures show measurements conducted at 140 kV using thresholds of 50 keV, 70 keV, and 90 keV. The tube voltage of 140 kV was chosen since it results in the highest CNRD according to previous sections. The top row of Fig. 5 shows the low energy bin, the second row the high energy bin, and the bottom row the iodine material map. All rows include a magnified region of interest including the right hepatic vein and the inferior caval vein. An increase of the threshold resulted in a noise reduction in the lower energy bin and in an increase in noise in the higher energy bin. I.e., using a threshold of 50 keV resulted in a noise level of 34 HU in the lower bin and in 23 HU in the higher bin. Increasing the threshold to 70 keV decreases noise in the lower bin to 25 HU and increases noise in the higher bin to 31 HU, while the noise is 24 HU and 45 HU in these bins at 90 keV. Since increasing the threshold increases the effective energy of the detected x-ray spectrum in both bins, contrast is reduced. This is particularly evident in the high energy bin and the iodine contrast in the larger vessels visualized therein. Similar to the results presented in previous sections, the best CNRD in the iodine material maps can be achieved using a threshold of 70 keV as 2.17 mGy^-1/2^ confirming the results presented in Figs. 3 and 4. Thresholds above or below this optimum show lower CNRDs of 1.30 mGy^-1/2^ using a threshold of 50 keV and 1.96 mGy^-1/2^ using a threshold of 90 keV.

## Discussion

Our study shows that the threshold of photon-counting detectors should be set according to the used tube voltage to ensure that image quality in material maps of iodine in terms of noise and CNRD is maximized. Since the investigated scan mode of the photon-counting detector always acquires two energy bins simultaneously, this also applies to standard examinations that are usually not intended as dual-energy examinations, but might be subject to a material decomposition retrospectively in case of accidental findings. Figure 2 illustrates that the relative contrast media ratio, a measure for the spectral separation between acquired bin data, is a function of the used threshold and increases with an increase thereof. Hence, if the noise in the low and high energy bin was constant, the best image quality would be achieved at the highest available threshold. However, since a change in threshold also affects the detected bin spectra and image noise, iodine CNRD shows distinct maxima as a function of tube voltage. This was illustrated using phantom measurements and in a post-mortem CT angiography study. Unlike in conventional dual-energy CT, the best image quality in the iodine maps was not achieved for a balanced image noise between bins, but for noise ratios between 0.6 and 0.8 (see Fig. 3). A comparison to iodine maps obtained using a conventional, energy-integrating dual-source dual-energy system revealed that the relative contrast media ratio of the investigated photon-counting detector is similar to the one observed in this system. However, given that the threshold cannot be changed without changing image noise and relative contrast media ratio, the image quality of the investigated photon-counting detector is slightly inferior to the reference system. This is consistent with the observations in the literature [29]. Other authors proposed the usage of dual-source photon-counting systems equipped with two photon-counting detectors to overcome this fact [30]. However, such systems do not yet exist and the cost-efficient future system might only be equipped with a single detector. The experiments presented herein assume a detector with two distinct energy bins. Other scan modes or detectors might provide even more simultaneously acquired energy bins. The used detector for example also provides a mode that acquires four bins simultaneously. However, R is not a suitable metric to describe the spectral separation between more than two energy bins. The design of suitable metrics for systems with more than two energy bins and the actual number of bins required to maximize e.g. the CNRD in iodine maps is a topic of ongoing research [31]. Photon-counting detectors not only allow for the simultaneous acquisition of multiple energy bins but also provide a variety of other benefits. Popular examples include but are not limited to a reduction in radiation and contrast media dose [32–34]. Hence, our experiments provide guidelines for threshold settings maximizing image quality in iodine maps, even if a dual-energy decomposition is only performed retrospectively.

## Abbreviations

CdTe: Cadmium telluride
CNR: Contrast-to-noise ratio
CNRD: Dose-normalized contrast-to-noise ratio
CT: Computed tomography
CTDI: Computed tomography dose index
DECT: Dual-energy CT
EI: Energy integrating
FOM: Field of measurement
FOV: Field of view
GOS: Gadolinium oxysulfide
PC: Photon counting
R: Relative contrast media ratio (RelCM)
RelNoise: Relative noise quotient
VMI: Virtual monochromatic imaging
VNC: Virtual non-contrast

## Declarations

### Guarantor

The scientific guarantor of this publication is Prof. Marc Kachelrieß.

### Statistics and biometry

No complex statistical methods were necessary for this paper.

### Methodology

• experimental
